# Statistical age determination of tree rings

**DOI:** 10.1371/journal.pone.0239052

**Published:** 2020-09-22

**Authors:** Martin Ricker, Genaro Gutiérrez-García, David Juárez-Guerrero, Margaret E. K. Evans

**Affiliations:** 1 Departamento de Botánica, Instituto de Biología, Universidad Nacional Autónoma de México, Mexico City, Mexico; 2 Departamento de Ciencias Ambientales y del Suelo, Instituto de Geología, Universidad Nacional Autónoma de México, Mexico City, Mexico; 3 Tree-Ring Building, University of Arizona, Tucson, Arizona, United States of America; University of Nevada, Reno, UNITED STATES

## Abstract

Dendrochronology, the study of annual rings formed by trees and woody plants, has important applications in research of climate and environmental phenomena of the past. Since its inception in the late 19^th^ century, dendrochronology has not had a way to quantify uncertainty about the years assigned to each ring (dating). There are, however, many woody species and sites where it is difficult or impossible to delimit annual ring boundaries and verify them with crossdating, especially in the lowland tropics. Rather than ignoring dating uncertainty or discarding such samples as useless, we present for the first time a probabilistic approach to assign expected ages with a confidence interval. It is proven that the cumulative age in a tree-ring time series advances by an amount equal to the probability that a putative growth boundary is truly annual. Confidence curves for the tree stem radius as a function of uncertain ages are determined. A sensitivity analysis shows the effect of uncertainty of the probability that a recognizable boundary is annual, as well as of the number of expected missing boundaries. Furthermore, we derive a probabilistic version of the mean sensitivity of a dendrochronological time series, which quantifies a tree’s sensitivity to environmental variation over time, as well as probabilistic versions of the autocorrelation and process standard deviation. A computer code in *Mathematica* is provided, with sample input files, as supporting information. Further research is necessary to analyze frequency patterns of false and missing boundaries for different species and sites.

## Introduction

**Fig 1:**
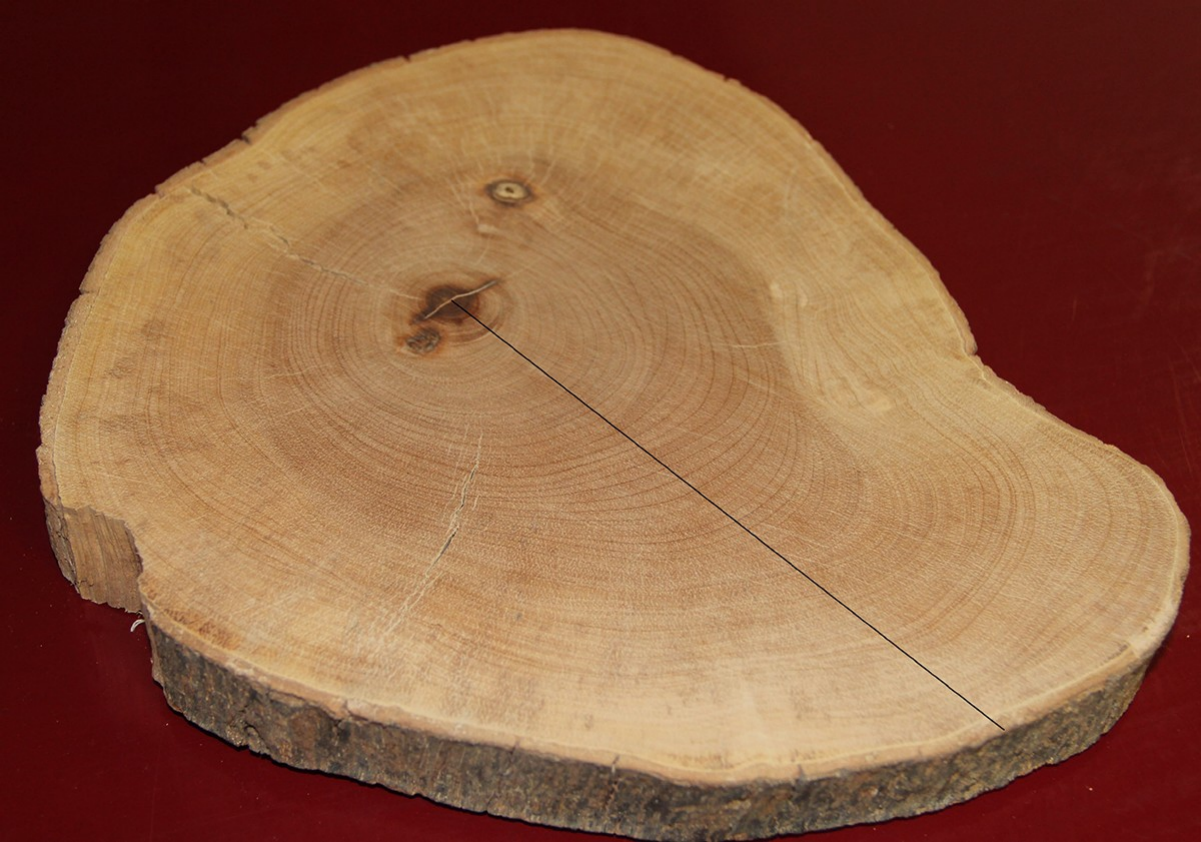
Cross-section of the bole of a tree, with observable tree rings (a tropical hardwood from near Coatzacoalcos, Veracruz, Mexico). Increment cores, such as the one in Fig 2, are taken with an increment borer from the bark towards the tree´s center (for example, along the black line), without destroying or severely harming the tree.

Tree growth generally follows an annual cycle, shaped by the dominant climatic cycle throughout a year. During reliably cold winters or dry periods, trees regularly cease to grow. If the climatic impact is sufficiently strong and extended, such growth variation results in annual, anatomically marked circular boundaries in a stem cross-section [1–4]. The circular bands between two boundaries, whose width can be measured, are called “tree rings” (Fig 1). The scientific field of dendrochronology (i.e., the study of time series of annual increments formed by trees and woody plants) started in the late 19^th^ century in Europe (chapter 1 in [5]). In dendrochronology, the ideal is to assign exact years to tree rings (page 96 in [6]). This is critical if the goal is to associate ring widths with climate variables or a particular event in a given year, such as an insect outbreak or volcanic eruption (pages 10-12 in [3]). For this reason, a great deal of attention has been paid in dendrochronology to verifying year assignments through crossdating [7]. In crossdating, the patterns of wide and narrow rings in samples from the same tree, as well as from several trees of the same area in parallel, are aligned, to correctly assign the corresponding year of formation. The idea is that the comparison amongst several samples reveals inconsistencies in individual samples (pages 11-15 in [6]). Once a calendar year has been assigned to a tree ring, a variety of statistical methods are used in dendrochronology to analyze the ring-width time series, for example, to extract climate signals with as little noise as possible [5].

As the field of dendrochronology grows, with more and more applications in tropical regions and research questions focused on global change ecology, it is not always easy or possible to meet the traditional standards for verifying year assignments (pages 12-14 in [3]). Firstly, a lack of variability in ring widths from one year to the next, known as “complacency”, can make it difficult to verify year assignments even by the usual means of crossdating, which fundamentally relies upon shared patterns of interannual variability in ring widths between time series (through visual inspection and high inter-series correlation). This is particularly a problem in “ecological” samples, i.e., from forest inventories or other efforts aimed at sampling forest ecosystems in an unbiased, representative manner. In these studies, the trees are chosen as random samples from a larger statistical population of trees in a forest ecosystem, implying that they are not necessarily growing on sites where one expects to see a high sensitivity of tree growth to climate. The goal in these ecological sampling designs may be to detect trends in forest growth or understand forest dynamics, rather than to analyze the climate of the past.

Secondly, an increment core may include some clearly identifiable ring boundaries, as well as other ring boundaries that are not clearly distinguishable, a situation that is frequent in the lowland tropics with less pronounced dry periods [8, 9]. In addition, there can be false and missing boundaries. For example, as part of Mexico’s national forest inventory we analyzed 1325 increment cores of different lengths, ranging from 3 to 14.5 cm (median length of 5.8 cm). The samples were taken from 190 hardwood species in 19 distinct Mexican States, at elevations from 0 to 1000 meters above sea level (median elevation of 223 m). Amongst these 1325 samples, 6.6% had clearly recognizable growth ring boundaries, 9.4% no visible boundaries, and 84.1% had ring boundaries that fell somewhere between these two extremes. The percentages refer to the “best” boundaries, when examining a complete core sample. In 31.4% of these samples, however, the recognizability of ring boundaries varied also along the (quite short) cores (unpublished data from coauthor DJG).

For these tropical collections, and in the case of unbiased, ecological samples, it would be valuable to develop a statistical approach that explicitly accounts for uncertainty in year assignments, rather than forcing a decision about the age of a ring boundary, or discarding such samples as useless (which may create bias in the statistical sample). Questions about forest growth, which translate into carbon sequestration, have taken on particular significance in the 21^st^ century, as we grapple with the need to store carbon in forests. There are many applications, where the assignment of an estimated age, relative to a base year, with a statistical confidence interval would be useful. For example, to create an average estimate of forest growth, or detect trends in growth, often one wants to estimate the past, not necessarily deterministic, growth curve of tree trunk radius as a function of time on a specific site. Even though the increment borer was invented by foresters as a tool to measure and compare past tree growth, a formal approach to statistical estimation of the corresponding tree-ring ages has never been developed in the field of dendrochronology. Here we introduce a method for probabilistic age estimation of tree-ring boundaries.

## Methods to obtain the input data

**Fig 2:**
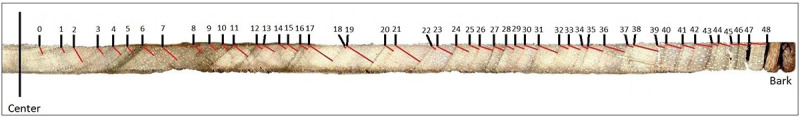
Increment core sample (labeled 81b), sampled from an *Alchornea latifolia* tree, with the enumeration of all ring boundaries. The length between the base boundary (0) and the last boundary (48) is 11.7 cm. Ring widths are marked with (red) diagonal lines that connect inner and outer boundaries, perpendicular to the ring’s inner boundary.

First, we briefly explain the development of our time-series data from an increment core. Samples were obtained with a Pressler increment borer, yielding increment cores (wood cylinders) of 5.08 mm in diameter and approximately 15 cm in length. The sample in Fig 2 was taken from a canopy tree of *Alchornea latifolia* in a closed forest of Jaguaroundi park, a protected area of Petróleos Mexicanos near Coatzacoalcos (Veracruz, Mexico). The park contains remnants of tropical wet forest, and has a pronounced dry season approximately from March to May. The first author (MR) had a collection permit for herbarium specimens and wood samples by Mexico’s SEMARNAT (Secretaría de Medio Ambiente y Recursos Naturales). In addition, the owner Petróleos Mexicanos had given permission to carry out the sampling.

Following standard dendrochronological sample preparation procedures, this increment core was sanded with progressively finer sandpaper. The image of the sample in Fig 2 was then taken under a stereo zoom microscope (Zeiss Axio Zoom.V16). The first visible ring boundary, i.e., closest to the center of the tree trunk (at left in Fig 2) is designated the “base boundary”, from which all other boundaries are counted. It receives the number 0. The ring between boundaries 0 and 1 gets the number 1; thus, the tree-ring’s number is the same as the number of its outer boundary. Towards the bark (on the right), a total of 49 boundaries are marked and counted (from 0 to 48). The last boundary (48) corresponds to the cambium, a band of a few cells width, from which wood (xylem) is formed to the inside, and bark (with phloem) to the outside.

Often, an increment core does not include the center of the tree (the pith), for example, when the pith is not where the sample taker expected it (in case of asymmetric growth); in that case the true age of the base boundary (0) is unknown. Furthermore, a tree has more growth rings at ground level than at greater heights (the difference being the number of years it took to reach the height at which the sample was taken). Therefore, when we refer to age, it is not the total age of the tree, but rather the *age of the wood formed after the base boundary*, whose absolute age may be unknown.

An additional input for our statistical algorithm is the time series of ring widths (radial increments). While ring widths are not needed as an input for the statistical age estimation presented below, they are necessary for growth curve graphs, and they are critical for the process of crossdating. Widths between inner and outer boundaries were measured along the connecting (red) line between any two boundaries, perpendicular to the ring’s inner boundary (Fig 2). We used the *Microsoft Paint* software to measure the pixel coordinates of the endpoints of each (diagonal) line that measures the width. The Pythagoras formula was then used to calculate the length of the line. For example, for tree ring 3: $\sqrt{{\left(246-233\right)}^{2}+{\left(178-164\right)}^{2}}=19.1$ pixels. Furthermore, the direct distance between boundary 0 and the end-boundary was measured to be 11.7 cm on the one hand, as well as 1738 pixels on the other, so that 19.1 pixels correspond to 0.129 cm.

Originally, we had collected samples from six *Alchornea latifolia* canopy trees in the same area, one of which is the sample in Fig 2, and attempted crossdating among them. The patterns of recognizable boundaries, however, were such that it was not clear which boundaries to consider annual in one sample and missing in the other, or false in the first place, i.e., crossdating would result in a highly subjective result. Consequently, this was a case, where a new method to assign tree ring boundaries probabilistically was necessary, if the samples were not to be discarded for growth analysis.

## Categories of tree-ring boundaries

When identifying and counting tree-ring boundaries, as those illustrated in Fig 2, we recognize four categories of ring boundaries. In the first two categories, the ring boundary is “**recognizable**”, i.e., characterized by recognizable changes in xylem (wood) cell characteristics associated with the slowing or complete cessation of tree growth, as caused by cold temperatures or lack of soil moisture. These boundaries may range from clearly marked to more diffuse and less distinguishable. Actually, it is a challenge to define objectively the term “recognizable boundary”. How blurred may it be? Ideally, a computer algorithm should be able to identify them with given sensitivity parameters.

There are two distinct, underlying causes for a recognizable ring boundary. One situation is that a boundary is “**annual**”, i.e., it marks the end of one year’s growth at a typical, annual climatic “interruption” (such as frost or dry period). Annual boundaries could also be called “regular”, “valid” or “true” boundaries. In temperate regions, these boundaries are formed before the frost period (“winter”). Second, recognizable boundaries can also be “**false**”. In that case, they are intra-annual boundaries, caused by stressful climatic periods (for example, lack of soil moisture) that divide an annual tree-ring artificially into at least two (page 47 in [6]). This gives rise to at least one additional ring that cannot be accounted for in dating. Frequently the term “false ring” is used, though it is more accurate to speak of a *false boundary*. Without crossdating or information on intra-annual climate variability, it may not be possible to distinguish annual and false boundaries.

The third and fourth categories are **missing boundaries** and **missing rings**, representing favorable versus unfavorable conditions for growth, respectively. A “missing” boundary may be suspected in unusually wide rings, and may be the result of a mild winter or a wetter-than-usual dry period in lowland tropical or less seasonal regions, i.e., two years of growth that appears as one year. A “missing ring” (page 21 in [3]) occurs when in extremely unfavorable (for example, dry) years a tree does not form wood in all or some portions of the bole. A missing ring can also be interpreted as two overlapping boundaries, and thus as a special case of a missing boundary.

Table 1 summarizes the four categories for the two boundary types “recognizable” and “missing”, together with the definition of the corresponding probabilities, as employed subsequently as input probabilities.

**Table 1:** Types of growth ring boundaries for assigning probabilities (0 < *P* ≤ 1).

Boundary type	Categories	Definition of input probability	Comment
Recognizable	1. Annual boundary; 2. False boundary	Probability *P* that a recognizable boundary represents an annual, regular growth phenomenon and not a false boundary. The probability for a false boundary is 1−*P*.	A boundary with input probability 1 is always an annual boundary.
Missing	1. Missing annual boundary; 2. Missing ring	Probability *P* that a missing annual boundary is expected at a specific location in a core sample. The number of missing boundaries has to be calculated from the expected frequency *F*, relative to the number of recognizable boundaries. For example, *F* = 0.04 expected missing boundaries times 50 boundaries in a sample = 2 missing boundaries. Placing 2 boundaries in the sample, each would have *P* = 1.	Either a boundary is missing because of continuous growth for more than one year, or a complete ring is missing because of a lack of growth. Even though these are opposite causes, *P* represents both situations, in terms of its effect of one less boundary. The input probability itself does not imply anything about the location of the missing boundary. A missing boundary, if present, corresponds necessarily to an annual boundary.

## Assigning years to ring boundaries probabilistically

Before using the data from Fig 2, we derive a general method for assigning years to ring boundaries, employing a small hypothetical example with assumed input probabilities. Table 2 shows the input. There are five boundaries, of which two are annual (*P* = 1), two are recognizable, but could be false (with two hypothetically different input probabilities), and there is also a missing boundary with a low input probability. The input probability that a recognizable boundary represents an annual boundary could vary if such boundaries are classified further, for example, by distinguishing them according to different anatomical features.

**Table 2:** Hypothetical input data and assignation of possible ages with probabilities for five boundaries.

Boundary number	Boundary type	Input probability (*P*) as defined in Table 1	Possible ages of boundary (0-4 years) and their calculated probabilities (*L*)				
			0	1	2	3	4
0	Annual (base boundary)	1	1				
1	Recognizable (annual or false)	0.7	0.3	0.7			
2	Annual	1		0.3	0.7		
3	Missing	0.2		0.24	0.62	0.14	
4	Recognizable (annual or false)	0.9		0.024	0.278	0.572	0.126

To illustrate how a probability distribution of ages accumulates for each ring boundary, we first consider the possible ages a given boundary could represent, without applying the input probabilities. Furthermore, for the moment ages will be considered integers. The base boundary is defined to have an age of 0 years. Boundary 1 is recognizable, so that it could be false (and thus still being associated with an age of 0 years) or annual and be 1 year old. Boundary 2 is annual, so that the two possible ages of boundary 1 become 1 year older. Boundary 3 is a missing one, so that potentially a year could be added, but otherwise the two possible previous ages would not change. Consequently, boundary 3 could be 1, 2, or 3 years old. Similarly, one more possible year is added for the last one, boundary 4. It is evident that only with annual boundaries or inclusion of missing boundaries with input probability 1, all previous ages become one year older. For boundaries with an input probability smaller than 1, all previous ages remain still possible, but one possible year is added.

Next, we apply the input probabilities. The base boundary (0) is defined to have an age of 0 years, and is the only boundary that is required to have an input probability of 1, i.e., to definitively exist as an annual boundary. If even the base boundary were doubtful (i.e., *P* < 1), the starting point for counting would be in question, which makes little sense. Furthermore, the base boundary could be the center of a tree trunk, in which case it is a point, rather than a boundary.

Boundary number 1 is annual with an input probability of 0.7. Therefore, with a probability of 1−0.7 = 0.3 it is false, and thus is still associated with an age of 0 years. However, with a probability of 0.7 it is annual, and in that case has an age of 1 year. Then follows the annual boundary 2, and the two probabilities remain the same, just at one year older. The missing boundary 3 follows. With probability 0.8, there is not a missing boundary, and with probability 0.2 the boundary demarks one year of growth (i.e., is annual). Under the first possibility, the probabilities 0.3 and 0.7 of boundary 2 are each multiplied with 0.8, which results in 0.24 and 0.56 that the ages of boundary 3 are the same as the ages of boundary 2. On the other hand, the probabilities 0.3 and 0.7 of boundary 2 are multiplied with 0.2 under the possibility that boundary 3 is annual, and is either 2 or 3 years old. This results in 0.06 and 0.14. The probabilities of the two possibilities are added, shifted by one year (Table 3):

**Table 3:** Boundary 3 in Table 2.

Boundary 3 in Table 2	1 year	2 years	3 years
For the case that there is no missing boundary:	0.3⋅(1−0.2) = 0.24	0.7⋅(1−0.2) = 0.56	0
For the case that there is a missing boundary:	0	0.3⋅0.2 = 0.06	0.7⋅0.2 = 0.14
Sum:	**0.24**	**0.62**	**0.14**

Note that the sum of these three probabilities adds up to 1 again. The same procedure is carried out for recognizable boundary 4 (Table 4):

**Table 4:** Boundary 4 in Table 2.

Boundary 4 in Table 2	1 year	2 years	3 years	4 years
For the case that the recognizable boundary is false:	0.24⋅(1−0.9) = 0.024	0.62⋅(1−0.9) = 0.062	0.14⋅(1−0.9) = 0.014	0
For the case that the recognizable boundary is annual:	0	0.24⋅0.9 = 0.216	0.62⋅0.9 = 0.558	0.14⋅0.9 = 0.126
Sum:	**0.024**	**0.278**	**0.572**	**0.126**

The example from Table 2 of calculating the probabilities of year assignments to each boundary can be generalized. First, we define the following symbols:

*i*: the boundary number;
*P*_*i*_: the input probability that boundary *i* represents an annual boundary;
*n*_*i*,*a*_: the number of possible ages (in years) for a given boundary;
*t*: the youngest possible age (in years) for a given boundary *i*;
*L*_*i*,*t*_: the calculated probability that boundary *i* is of age *t* (in years); the letter “L” is used for “likelihood” to distinguish it from “P” (but has nothing to do here with a likelihood function).

Generalizing the example from Table 2, for any number of calculated probabilities *n*_*i*,*a*_, the recursive formula to calculate the probabilities *L*_*i*+1_ from *L*_*i*_ becomes

$$n_{i,a}=1\Rightarrow \left\{ \begin{array}{l} L_{i+1,t}=1-P_{i+1},\\ L_{i+1,t+1}=P_{i+1}, \end{array}\right.$$ (1A)

$$n_{i,a}=2\Rightarrow \left\{ \begin{array}{l} L_{i+1,t}=L_{i,t}\cdot \left(1-P_{i+1}\right),\\ L_{i+1,t+1}=L_{i,t+1}+P_{i+1}\cdot \left(L_{i,t}-L_{i,t+1}\right),\\ L_{i+1,t+2}=L_{i,t+1}\cdot P_{i+1}, \end{array}\right.$$ (1B)

$$n_{i,a}>2\Rightarrow \left\{ \begin{array}{l} L_{i+1,t}=L_{i,t}\cdot \left(1-P_{i+1}\right),\\ L_{i+1,t+1}=L_{i,t+1}+P_{i+1}\cdot \left(L_{i,t}-L_{i,t+1}\right),\\ L_{i+1,t+2}=L_{i,t+2}+P_{i+1}\cdot \left(L_{i,t+1}-L_{i,t+2}\right),\\ \ldots ,\\ L_{i+1,t+n_{i,a}-1}=L_{i,t+n_{i,a}-1}+P_{i+1}\cdot \left(L_{i,t+n_{i,a}-2}-L_{i,t+n_{i,a}-1}\right),\\ L_{i+1,t+n_{i,a}}=L_{i,t+n_{i,a}-1}\cdot P_{i+1}. \end{array}\right.$$ (1C)

Note that with *P*_*i*+1_ = 1, *L*_*i*+1,*t*_ becomes always 0, i.e., there is no probability that the next boundary remains at the same year. For example, for the transition from *i* = 3 to *i* = 4 in Table 2, *n*_*i*,*a*_ = 3, so that (1C) applies; one has *i*= 3 and *i* = 1:

$$\begin{array}{l} L_{3+1,1}=L_{3,1}\cdot \left(1-P_{3+1}\right),\\ L_{3+1,1+1}=L_{3,1+1}+P_{3+1}\cdot \left(L_{3,1}-L_{3,1+1}\right),\\ L_{3+1,1+2}=L_{3,1+2}+P_{3+1}\cdot \left(L_{3,1+1}-L_{3,1+2}\right),\\ L_{3+1,1+3}=L_{3,1+3-1}\cdot P_{3+1}, \end{array}$$

which parameterized yields the numbers from Table 2:

$$\begin{array}{l} L_{4,1}=L_{3,1}\cdot \left(1-P_{4}\right)=0.24\cdot \left(1-0.9\right)=0.024,\\ L_{4,2}=L_{3,2}+P_{4}\cdot \left(L_{3,1}-L_{3,2}\right)=0.62+0.9\cdot \left(0.24-0.62\right)=0.278,\\ L_{4,3}=L_{3,3}+P_{4}\cdot \left(L_{3,2}-L_{3,3}\right)=0.14+0.9\cdot \left(0.62-0.14\right)=0.572,\\ L_{4,4}=L_{3,3}\cdot P_{4}=0.14\cdot 0.9=0.126. \end{array}$$

A computer code in *Mathematica*, to carry out these calculations for a whole tree-ring time series, is provided as supporting information.

Note that for the case where the *P*_*i*_ for all boundaries (except the base boundary) are equal, one can apply the formulas for the binomial distribution (chapter 5.2 in [10]). To calculate the probabilities for boundary 4, one defines *Q* = 1−*P*, expands (*Q*+*P*)^3^, separates the summands, and substitutes *Q* with 1−*P*, which yields (1−*P*)^3^, 3⋅*P*⋅(1−*P*)^2^, 3⋅*P*^2^⋅(1−*P*), and *P*^3^. Substituting, for example, with *P* = 0.83 results in the probabilities for the ages of 1 to 4 years of 0.005, 0.072, 0.351, and 0.572. However, we will not treat the binomial distribution further here. Instead, we continue with the general case of varying input probabilities (*P*_*i*_) among boundaries.

## Mean ages and standard deviations

First we calculate the mean (or expected) age ${\bar{A}}_{i}$ for each boundary *i*. If for a given boundary number *i*, its true age *A*_*i*_ is considered a random variable of discrete type, the formula for the expectation *E*(*A*_*i*_) is (page 132 in [11]):

$${\bar{A}}_{i}=E\left(A_{i}\right)=\sum_{j=0}^{n_{i,a}-1}\left[\left(t+j\right)\cdot L_{i,t+j}\right].$$ (2)

For boundary 3, for the example in Table 2 on gets ${\bar{A}}_{3}=\sum_{j=0}^{3-1}\left[\left(1+j\right)\cdot L_{3,1+j}\right]=1\cdot L_{3,1}+2\cdot L_{3,2}+3\cdot L_{3,3}=1\cdot 0.24+2\cdot 0.62+3\cdot 0.14=1.9$ years. Next, the variance of *A*_*i*_ is calculated (page 133 in [11]):

$$s_{A_{i}}^{2}=E\left[{\left(A_{i}-{\bar{A}}_{i}\right)}^{2}\right]=E\left(A_{i}^{2}\right)-{\bar{A}}_{i}^{2}=\sum_{j=0}^{n_{i,a}-1}\left[{\left(t+j\right)}^{2}\cdot L_{i,t+j}\right]-{\bar{A}}_{i}^{2},$$ (3)

where *E* refers again to the expectation. In the example, with (3) one gets $s_{A_{3}}^{2}=\sum_{j=0}^{3-1}\left[{\left(1+j\right)}^{2}\cdot L_{3,1+j}\right]-{\bar{A}}_{3}^{2}=1^{2}\cdot L_{3,1}+2^{2}\cdot L_{3,2}+3^{2}\cdot L_{3,3}-1.72^{2}=1\cdot 0.24+4\cdot 0.62+9\cdot 0.14-1.9^{2}=0.37$. The standard deviation is $s_{A_{i}}=\sqrt{s_{A_{i}}^{2}}$, which in the example is $\sqrt{0.37}\approx$ 0.608. If the input probabilities *P*_*i*_ in (1A)–(1C) are considered parametric (without uncertainty), then $\bar{A}$ and $s_{A_{i}}^{2}$ represent the true (not sample) parameters of a statistical population (typically denoted as *μ* and *σ*^2^).

In this way, one calculates ${\bar{A}}_{1}$, ${\bar{A}}_{2}$, ${\bar{A}}_{3}$, etc. How does the estimated (expected) age develop, when advancing from one tree-ring to the next, and the annual nature of the boundaries is probabilistic?

*Theorem 1*: The age in a tree-ring time series advances by an annual proportion that is numerically equal to the input probability *P*_*i*_ that the putative boundary *i* of a tree-ring is truly annual; for example, a tree ring that represents an annual boundary with input probability 0.8 increases the cumulative age by 0.8 years.

*Proof*: Consider two calculated probabilities *L*_*i*,*t*_ and *L*_*i*,*t*+1_ for ring-boundary *i* to have formed either at age *t* or *t*+1, respectively. The probability that ring-boundary *i*+1 represents an annual boundary is *P*_*i*+1_. Then the calculated probabilities for boundary *i*+ 1 without a shift in age become *L*_*i*,*t*_⋅(1−*P*_*i*+1_) and *L*_*i*,*t*+1_⋅(1−*P*_*i*+1_), and with a shift in age *L*_*i*,*t*_⋅*P*_*i*+1_ and *L*_*i*,*t*+1_⋅*P*_*i*+1_. Summing as in Table 2 results in *L*_*i*,*t*_⋅(1−*P*_*i*+1_) for age *t*, *L*_*i*,*t*+1_⋅(1−*P*_*i*+1_)+*L*_*i*,*t*_⋅*P*_*i*+1_ for age *t*+1, and *L*_*i*,*t*+1_⋅*P*_*i*+1_ for age *t*+2. According to (2), the corresponding expected age becomes:

$${\bar{A}}_{i+1}=t\cdot L_{i,t}\cdot \left(1-P_{i+1}\right)+\left(t+1\right)\cdot \left(L_{i,t+1}\cdot \left(1-P_{i+1}\right)+L_{i,t}\cdot P_{i+1}\right)+\left(t+2\right)\cdot L_{i,t+1}\cdot P_{i+1},$$

which simplifies to

$${\bar{A}}_{i+1}=L_{i,t}\cdot \left(P_{i+1}+t\right)+L_{i,t+1}\cdot \left(1+P_{i+1}+t\right).$$

With two calculated probabilities, one has *L*_*i*,*t*_+*L*_*i*,*t*+1_ = 1⇒*L*_*i*,*t*_ = 1−*L*_*i*,*t*+1_, so that one can substitute *L*_*i*,*t*_ and simplify further:

$${\bar{A}}_{i+1}=t+L_{i,t+1}+P_{i+1}.$$

The expected age (${\bar{A}}_{i+1}$) is the age to start with (*t*), plus the calculated probability of the previous boundary (*L*_*i*,*t*+1_), plus the probability of the subsequent boundary (*P*_*i*+1_).

## Estimation of the input probabilities for annual and missing boundaries

The probability for a recognizable boundary to be an annual (and not a false) boundary, and the probability that recognizable boundaries are accompanied by missing boundaries (Table 1) are estimated from the frequencies with which these different boundaries are found in trees. If one knows that there are 80 annual and 20 false boundaries in a sample of 100 recognizable boundaries, then the probability of an annual boundary is 0.8. If one knows that in addition there is one missing boundary, then the frequency *F* of a recognizable boundary being accompanied by a missing boundary is 1/100 = 0.01.

The frequencies of non-annual boundaries will vary according to species, site, habitat, and other factors. Missing rings vary among species, genera, and also the individual tree’s position in the forest canopy. In a synthesis of locally missing growth rings across the Northern Hemisphere, based on 2359 publicly available tree ring-width time series, missing rings were most common in *Pinus* (0.8% of all rings missing) and *Pseudotsuga* (0.6%), occasional in *Larix* (0.2%), and rare in *Picea* (0.03%) and *Quercus* (0.01%) [12].

Suppressed trees may not form wood, and the corresponding ring will be missing, a situation observed in understory trees from temperate regions. In a study of 95 stem disks of *Acer saccharum* from Wisconsin (USA), the number of ring anomalies was inversely related to growth rate and vigor of the trees; the mean percentage of ring anomalies was 16.2% in overtopped trees, and all of the overtopped trees had partial or missing rings [13]. Similarly, suppressed trees of *Fagus sylvatica* in Southern Sweden presented 36 missing rings in a time series of 226 years [14]. In *Pinus banksiana* in Michigan (USA), the trees’ early years were more likely to contain false rings [15].

In the lowland tropics, however, boundaries do not form because of reliably cold winters; rather they form in response to dry periods, which may or may not be reliably dry. Interannual variability in intensity and length of the dry period can cause apparently continuous growth over two years without a boundary (a “missing boundary”). We have not been able to find literature that reports frequencies of this latter phenomenon.

We hypothesize that in general, canopy trees in humid tropical regions will present a relatively large number of false boundaries versus a relatively small number of missing rings or missing boundaries, because of repeated interruptions of growth from short dry periods. However, we do not know how much this might vary across species and trees in different canopy positions within species.

Several methods can be used to determine “true” frequencies of annual (versus false) and missing boundaries:

1. Samples could be crossdated from the same tree species in another region, where crossdating works, and assuming that the environmental conditions are representative [16]. Alternatively, one could crossdate samples from another tree species in the same or at least a similar region, assuming that the result from the other species is representative.
2. Intra-annual growth, and its relation with climate during at least one year, can be studied either with dendrometers or more accurately with micro-cores [17].
3. Tree ages and ring ages can be determined from core samples by radiocarbon (^14^C) dating [16, 18, 19], though in [19] the need to develop ^14^C calibration curves for South America is noted to improve accuracy.
4. A more recent method to estimate tree ages, and ring or annual-increment ages (in case of no distinct annual rings), is the use of stable isotopes of oxygen (δ^18^O), and to a lesser degree also carbon (δ^13^C) along the radius with samples from high-resolution wood sampling [20–23]. The method detects primarily interannual changes in wet season precipitation [20], and could serve to develop a number of representative input probabilities for different species and environmental situations.

The objective here was to have a reasonable (though not necessarily very accurate) estimate for the input probabilities to be able to demonstrate the method. One of the coauthors (GGG) had crossdated core samples from *Pinus oocarpa* in the Los Tuxtlas region of Veracruz, at a distance of about 50 km from Parque Jaguaroundi, with a similar climate. Analyzing 1861 tree rings from 35 core samples of 29 trees, there were 313 false rings (16.8%) and 2 missing rings (pages 120-121 in [24]). The corresponding probability that a recognizable boundary is an annual boundary is

$$\frac{1861-313}{1861}\approx 0.83.$$

The probability that a recognizable boundary is accompanied by a missing boundary is 2/1861≈0.001. Since for 48 tree rings 48⋅0.001≈0.05, i.e., far below 1 expected missing boundary, we will not consider missing boundaries in this example from Fig 2 until the sensitivity analysis.

The probabilistic situation of missing boundaries is fundamentally different from recognizable boundaries, because one cannot assign a probability to an observed boundary, but rather one has to calculate a number of expected boundaries. If the probability for a missing boundary were around 0.02, one would assume one missing boundary in our sample (48⋅0.02 = 0.96). If crossdating does not provide clues, and without further analysis about climate fluctuations, one would have to place it at a reasonable location on the core sample, probably at the midpoint of an unusually wide ring. There is no probability available for the correctness of the chosen location on the core sample, i.e., the uncertainty associated with inserting a missing ring in any particular position in the time series of rings. One can check if there are diffuse boundaries that are less-than-recognizable. If one assigns the exact expected number of missing boundaries, then each one has an input probability of 1. If there are several possible locations on the core sample, and thus one assigns more missing boundaries than expected, then the input probability for each missing boundary decreases accordingly. For example, if there were 2 expected missing boundaries, but 5 rings on the core samples are suspected to contain missing boundaries, then each of 5 assigned missing boundaries would have an input probability of 2/5 = 0.4.

## Confidence curves

**Fig 3:**
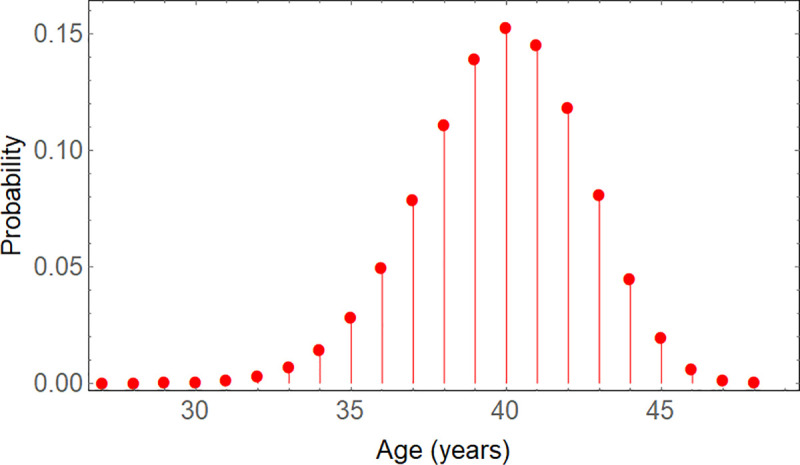
Discrete probability density function of the age of boundary 48, for ages above 26 years. The distribution is limited to the range of 0 to 48 years. The estimated mean (or expected) age is 39.84 years. This is equal to the sum of input probabilities after the base boundary, here 0.83⋅48, as expected from Theorem 1.

Now the expected ages of the ring boundaries in the sample from Fig 2 can be evaluated in detail. Only the base boundary is certainly annual, whereas all subsequent boundaries are recognizable with an estimated input probability of 0.83 to be annual, and there are no missing boundaries assumed (see previous section). Other possible scenarios will be shown in the sensitivity analysis below. Once the input probabilities for all boundaries are estimated, it becomes possible to examine the statistical distribution of possible ages for a given tree-ring boundary. Fig 3 shows the result for the last boundary (number 48), with the input probabilities for 49 different ages, for ages above 26 years. The probability density function in Fig 3 is discrete, i.e., for integer years. It is a truncated statistical distribution, within an interval from the smallest to the largest possible age of a given boundary. For each boundary, its statistical distribution will be used to calculate confidence intervals for the age it may have.

**Fig 4:**
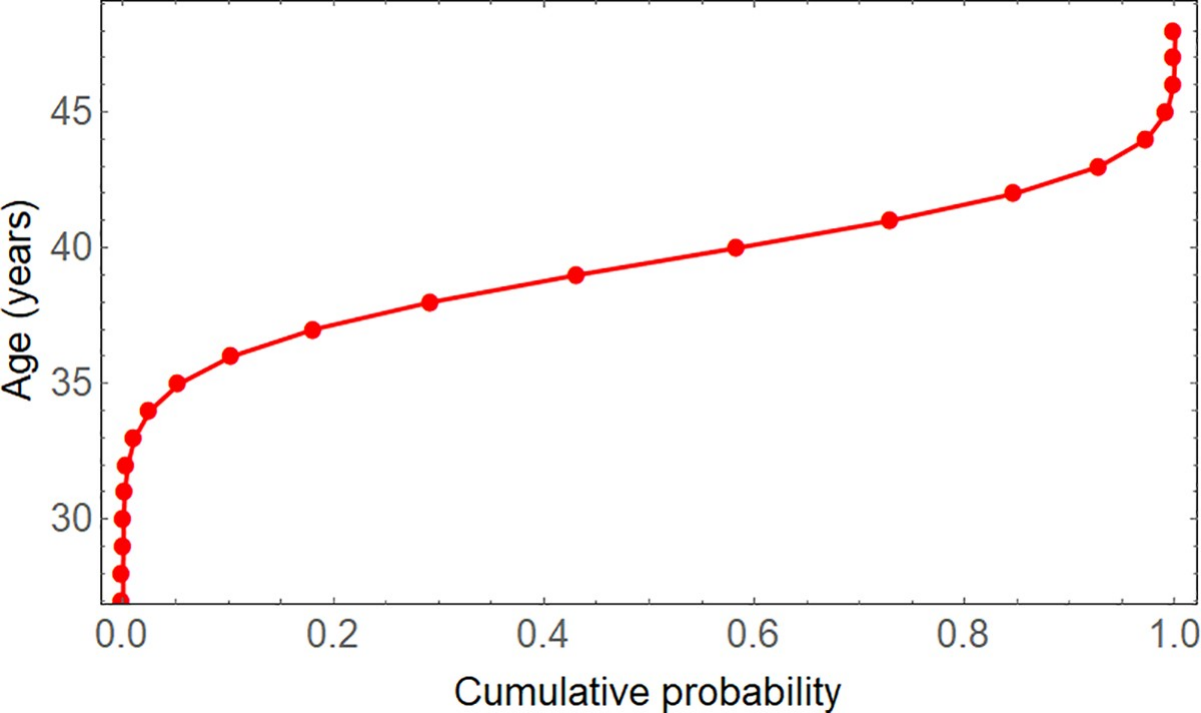
The inverse cumulative distribution function for boundary 48. The calculated probabilities are piecewise linearly connected to get a continuous function. It serves to find confidence limits for the estimated age, for a 95% confidence interval at 2.5% and 97.5%. The age goes downwards to 0, and the cumulative probability on the left also towards 0.

We are interested in calculating (95%-)confidence intervals for the expected age ${\bar{A}}_{i}$, and subsequently deriving confidence curves. Though we have formulated the problem in terms of annual intervals between ring boundaries (because of annual seasonality), in principle time is a continuous variable. It makes sense to consider non-integer confidence limits for estimated ages of tree rings, and a continuous probability distribution function is necessary to calculate them with accuracy. The probabilities of different ages for a given tree ring are added cumulatively, and the corresponding ages plotted as a continuous function of those cumulative probabilities, with linear interpolation. Fig 4 shows the continuous inverse cumulative distribution function (CDF), where the cut-off age is shown as a function of the cumulative probability, corresponding to the discrete probability density function of Fig 3. From this interpolated piecewise linear function, confidence limits are determined by searching for the ages that a boundary would have with a probability of 2.5% and 97.5%, respectively. The only peculiarity here is that for the first possible age of a given boundary, the probability could already be larger than 2.5% (at early ages), so that one cannot interpolate to the left, and a corresponding confidence limit does not exist. For example, in Table 2 boundary 2 is at a minimum 1 year old with 30% probability; so a confidence limit would be at less than the lower limit of 1 year, which does not make sense. In these cases, the lower confidence limit can be substituted by the lower bound, i.e., here 1 year.

Table 5 shows the resulting statistics for the possible ages of the first and last boundaries of the data from Fig 2. The cumulative age increases according to the input probabilities (as predicted by Theorem 1), and the number of possible ages increases each time by one year in the second column. Furthermore, for the first boundaries the difference between the range of possible ages (column 2) and the 95% confidence limits (last two columns) is trivial, but for higher-numbered boundaries, the confidence interval is clearly smaller than the age range.

**Table 5:** Statistics of age assignment for a subset of the ring boundaries from Fig 2.

Boundary number (input probability)	Range of possible ages	Mean age ($\bar{A}$)	Standard deviation ($s_{A_{i}}$)	Lower 95% confidence limit of age (or lowest possible age)	Upper 95% confidence limit of age
0 (1)	0-0	0	0	0	0
1 (0.83)	0-1	0.83	0.376	0	0.97
2 (0. 83)	0-2	1.66	0.531	0	1.96
3 (0. 83)	0-3	2.49	0.651	0.28	2.96
…	…	…	…	…	…
46 (0. 83)	0-46	38.18	2.548	32.34	42.40
47 (0. 83)	0-47	39.01	2.575	33.15	43.26
48 (0. 83)	0-48	39.84	2.602	33.96	44.10

**Fig 5:**
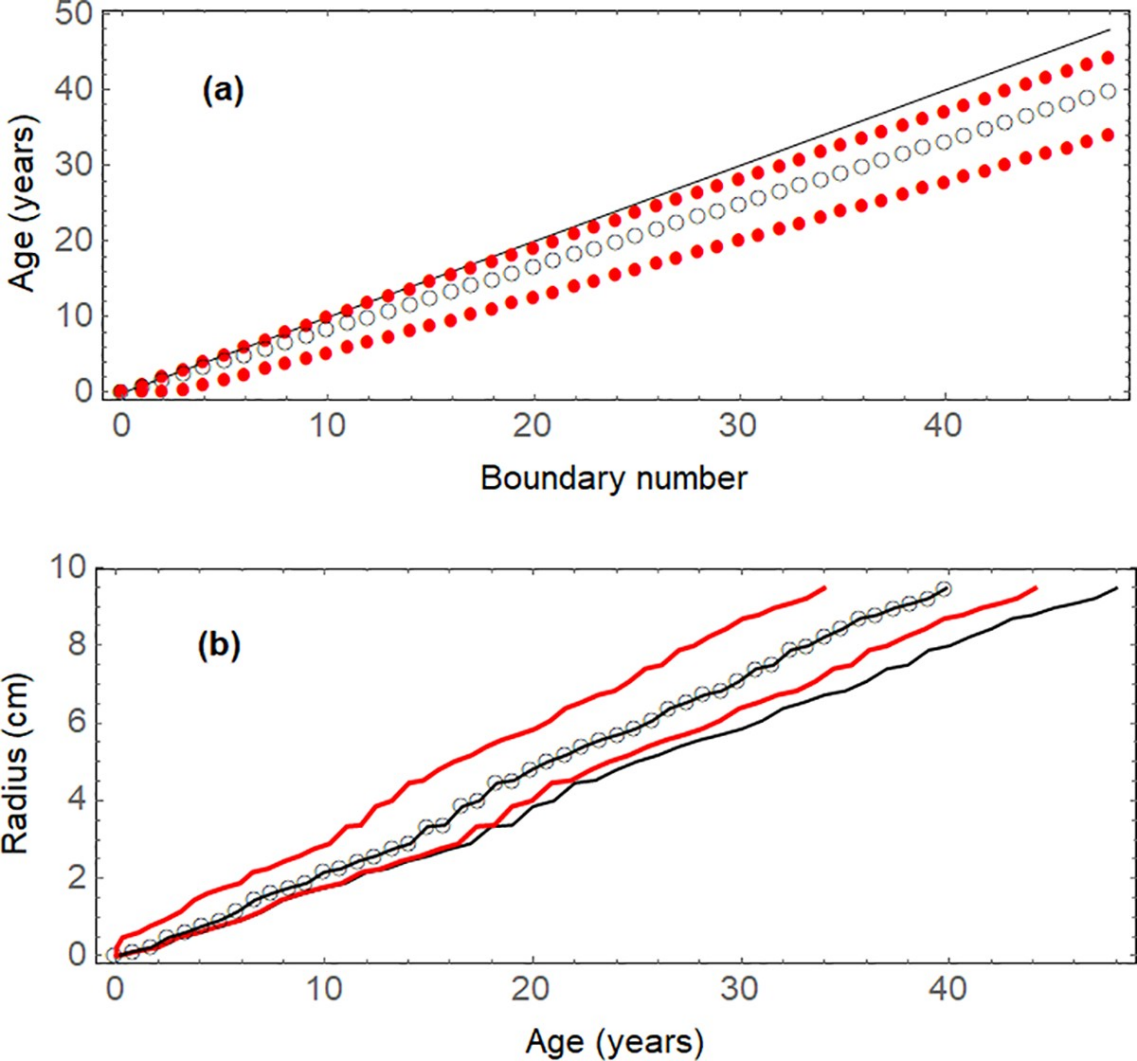
Uncertainty about ages of tree-ring boundaries. (a) estimated ages in years with 95% confidence limits (red) points; the points cannot be above the (black) diagonal line, where the age is equal to the boundary number; (b) radius as a function of estimated age, with 95% confidence limits (points are linearly interpolated). While the (black) thinner, lower line represents the upper age limit, the lower age limit is given by the *Y*-axis.

Estimated ages of all ring boundaries, with 95% confidence intervals, are shown in Fig 5A. Note that the upper limit of possible ages lies on the 45° diagonal, because the maximum age is always equal to the boundary number. Fig 5B subsequently shows cumulative radius, based on the radial increments, as a function of age (linearly interpolated), together with confidence curves that take into account the uncertainty about year assignments. In contrast to usual age-radius curves, these curves are delimited horizontally at the maximum measured radius, not vertically at the maximum measured age. Furthermore, there can be vertical lines, connecting several radii that are associated with the same age.

## Probabilistic mean sensitivity, autocorrelation, and process standard deviation of a tree-ring series

An additional parameter of interest in dendrochronology and dendroclimatology is the *mean sensitivity of a tree-ring series*. Douglass introduced it as “the average percentage increment from year to year without regard to sign. A tree of high sensitivity is more datable and more likely to make a contribution to our studies than a tree with a complacent series of rings” (page 9 in [25]). Fritts defined the mean sensitivity with the following formula (page 258 in [3]):

$$ms=\frac{1}{n_{w}-1}\cdot \sum_{i=1}^{n_{w}-1}Abs\left[\frac{2\cdot \left(w_{i+1}-w_{i}\right)}{w_{i+1}+w_{i}}\right],$$ (4)

where *w*_*i*_ is the width (radial increment) of ring *i*, *n*_*w*_ is the number of radial increments in the time series, and *Abs* refers to the absolute value. For a sample with *n*_*b*_ boundaries, there are *n*_*w*_ = *n*_*b*_−1 widths (radial increments). Note that (4) calculates the change of the width between two rings *w*_*i*+1_−*w*_*i*_ relative to the two rings’ mean width (*w*_*i*_+*w*_*i*+1_)/2. The value of *ms* ranges from 0, when all widths are the same, to 2, when rings are alternating (theoretically) between zero and positive width.

We consider now

$$X_{i}=Abs\left[\left(2\cdot \left(w_{i+1}-w_{i}\right)\right)/\left(w_{i+1}+w_{i}\right)\right]$$

to be a random variable. A fundamental property in statistics is that “the expectation of a sum of random variables is the sum of the expectations, no matter how the two variables are related” (page 69 in [26]): *E*(*X*_1_+*X*_2_+…) = *E*(*X*_1_)+*E*(*X*_2_)+…. Furthermore, with the logic from (2), *E*(*X*_*i*_) = *X*_*i*_⋅*P*_*i*+1_, because *P*_*i*+1_ represents the input probability that the boundary between the ring with width *w*_*i*_ and the next ring with width *w*_*i*+1_ is annual. Consequently, the probabilistic mean sensitivity of a tree-ring series $\tilde{ms}$ becomes:

$$\tilde{ms}=\frac{1}{n_{w}-1}\cdot \sum_{i=1}^{n_{w}-1}Abs\left[P_{i+1}\cdot \frac{2\cdot \left(w_{i+1}-w_{i}\right)}{w_{i+1}+w_{i}}\right],$$ (5)

where the probabilistic version of the variable is indicated by a tilde. The division in (5) of the expected sum *E*(*X*_1_+*X*_2_+…) by *n*_*w*_−1 results in a mean expected value for *X*_*i*_.

Since *σ*^2^ = *E*(*X*^2^)−*μ*^2^ (page 133 in [11]), the estimated variance is $s_{\tilde{ms}}^{2}=E\left(X_{i}^{2}\right)-{\left(\tilde{ms}\right)}^{2},$ or

$$s_{\tilde{ms}}^{2}=\frac{1}{n_{w}-1}\cdot \sum_{i=1}^{n_{w}-1}\left[P_{i+1}\cdot {\left(\frac{2\cdot \left(w_{i+1}-w_{i}\right)}{w_{i+1}+w_{i}}\right)}^{2}\right]-{\left(\tilde{ms}\right)}^{2}.$$ (6)

The corresponding standard error of the mean is $SE_{\tilde{ms}}=\sqrt{s_{\tilde{ms}}^{2}/n_{w}}$ (page 139 in [10]).

The mean sensitivity of a tree-ring series has been used as an indicator for over 80 years, but has also been criticized. Arguments against using it have been that the mean sensitivity, as defined in (4), is proportional to the standard deviation of the ring width (or measured variable), except in cases with strong autocorrelation [27], and that it is ambiguous, because it does not separate variance and autocorrelation [28]. If this critique is warranted is debatable: To think of mean sensitivity in terms of a combination of variance (or standard deviation) and autocorrelation seems reasonable. Nevertheless, we take up the recommendation from [27], and provide also formulas for the probabilistic standard deviation and autocorrelation of the time series of ring widths.

The first-order autocorrelation for a stationary stochastic process is defined, here for the ring widths, as (page 31 in [29]):

$$r_{ac1}=\frac{\sum_{i=1}^{n_{w}-1}\left[\left(w_{i}-\bar{w}\right)\cdot \left(w_{i+1}-\bar{w}\right)\right]}{\sum_{i=1}^{n_{w}}\left[{\left(w_{i}-\bar{w}\right)}^{2}\right]}.$$ (7)

Strictly speaking, the ring widths should be standardized, before calculating the autocorrelation, to account for the expected change of width with the tree’s age and thus comply with the assumption of a stationary stochastic process (i.e., constant random noise around a constant mean path) (page 24 in [29]). A linear regression of the widths from Fig 2, however, results in a nonsignificant regression coefficient of 0.00048 and a reasonably constant variance with time, so that standardization is not important here.

The variance of the time series of ring widths is (page 28 in [30]):

$$s_{ts}^{2}=\frac{1}{n_{w}}\cdot \sum_{i=1}^{n_{w}}{\left(w_{i}-\bar{w}\right)}^{2},$$ (8)

where in contrast to the usual formula for the variance the division is by *n*_*w*_ (not *n*_*w*_−1). Furthermore, the variance of the first-order autoregressive (Markov) process is $s_{proc}^{2}=s_{ts}^{2}/\left(1-r_{ac1}^{2}\right)$ (page 61 in [29]), and consequently the process standard deviation becomes:

$$s_{proc}=\sqrt{\frac{s_{ts}^{2}}{1-r_{ac1}^{2}}}.$$ (9)

The process standard deviation could be a useful, alternative indicator of the mean sensitivity, as it takes into account both variance and autocorrelation.

Next, we wish to include the input probabilities of the ring boundaries. If one considers ${\left(w_{i}-{\bar{w}}_{i}\right)}^{2}$ or alternatively $\left(w_{i}-{\bar{w}}_{i}\right)\cdot \left(w_{i+1}-{\bar{w}}_{i}\right)$ to be a random variable, then one can follow the same logic as above for deriving (5), and insert the input probabilities correspondingly in (7) and (8), to get a probabilistic autocorrelation and time series’ variance:

$${\tilde{r}}_{ac1}=\frac{\sum_{i=1}^{n_{w}-1}\left[P_{i+1}\cdot \left(w_{i}-\bar{w}\right)\cdot \left(w_{i+1}-\bar{w}\right)\right]}{\sum_{i=1}^{n_{w}}\left[P_{i}\cdot {\left(w_{i}-\bar{w}\right)}^{2}\right]},$$ (10)

$${\tilde{s}}_{ts}^{2}=\frac{1}{n_{w}}\cdot \sum_{i=1}^{n_{w}}\left[P_{i}\cdot {\left(w_{i}-\bar{w}\right)}^{2}\right].$$ (11)

With (10) and (11) in (9), one gets the probabilistic version of the process standard deviation:

$${\tilde{s}}_{proc}=\sqrt{\frac{{\tilde{s}}_{ts}^{2}}{1-{\tilde{r}}_{ac1}^{2}}}.$$ (12)

The difference between *s*_*proc*_ from (9) and ${\tilde{s}}_{proc}$ from (12) is that *s*_*proc*_ calculates a standard deviation for widths of varying size, whose overall number is certain. In contrast, ${\tilde{s}}_{proc}$ adjusts the expectation for the standard deviation, taking into account uncertainty if a given width corresponds to an annual ring. Table 6 summarizes formulas (4) to (12).

**Table 6:** Standard and probabilistic formulas for mean sensitivity, autocorrelation, and process standard deviation.

Concept	Standard formula	Probabilistic formula
Mean sensitivity of a dendrochronological time series	$\begin{array}{l} ms=\frac{1}{n_{w}-1}\\ \cdot \sum_{i=1}^{n_{w}-1}Abs\left[\frac{2\cdot \left(w_{i+1}-w_{i}\right)}{w_{i+1}+w_{i}}\right] \end{array}$	$\begin{array}{l} \tilde{ms}=\frac{1}{n_{w}-1}\\ \cdot \sum_{i=1}^{n_{w}-1}Abs\left[P_{i+1}\cdot \frac{2\cdot \left(w_{i+1}-w_{i}\right)}{w_{i+1}+w_{i}}\right] \end{array}$
Variance of the mean sensitivity	No	$\begin{array}{l} s_{\tilde{ms}}^{2}=\frac{1}{n_{w}-1}\\ \cdot \sum_{i=1}^{n_{w}-1}\left[P_{i+1}\cdot {\left(\frac{2\cdot \left(w_{i+1}-w_{i}\right)}{w_{i+1}+w_{i}}\right)}^{2}\right]\\ -{\left(\tilde{ms}\right)}^{2} \end{array}$
Autocorrelation for a stationary stochastic process	$r_{ac1}=\frac{\sum_{i=1}^{n_{w}-1}\left[\begin{array}{l} \left(w_{i}-\bar{w}\right)\\ \cdot \left(w_{i+1}-\bar{w}\right) \end{array}\right]}{\sum_{i=1}^{n_{w}}{\left(w_{i}-\bar{w}\right)}^{2}}$	${\tilde{r}}_{ac1}=\frac{\sum_{i=1}^{n_{w}-1}\left[\begin{array}{l} P_{i+1}\cdot \left(w_{i}-\bar{w}\right)\\ \cdot \left(w_{i+1}-\bar{w}\right) \end{array}\right]}{\sum_{i=1}^{n_{w}}\left[P_{i}\cdot {\left(w_{i}-\bar{w}\right)}^{2}\right]},$
Variance of a time series	$s_{ts}^{2}=\frac{1}{n_{w}}\cdot \sum_{i=1}^{n_{w}}{\left(w_{i}-\bar{w}\right)}^{2}$	${\tilde{s}}_{ts}^{2}=\frac{1}{n_{w}}\cdot \sum_{i=1}^{n_{w}}\left[P_{i}\cdot {\left(w_{i}-\bar{w}\right)}^{2}\right].$
Process standard deviation of a first-order autoregressive (Markov) process	$s_{proc}=\sqrt{\frac{s_{ts}^{2}}{1-r_{ac1}^{2}}}$	${\tilde{s}}_{proc}=\sqrt{\frac{{\tilde{s}}_{ts}^{2}}{1-{\tilde{r}}_{ac1}^{2}}}.$

Using the time series from Fig 2, the probabilistic estimate of mean sensitivity with (5) is $\tilde{ms}=$ 0.511 (without any unit), and its standard error with (6) is $SE_{\tilde{ms}}=$ 0.0652 (*n* = 48). This compares to a traditional (non-probabilistic) mean sensitivity of 0.616, calculated with (4). Confidence limits can be estimated under the assumption that the mean sensitivity has an approximately normal distribution (invoking the central limit theorem). The 95% confidence limits are calculated consequently as $\tilde{ms}-1.96\cdot SE_{\tilde{ms}}$ and $\tilde{ms}+1.96\cdot SE_{\tilde{ms}}$ (page 158 in [10]). For the example in Fig 2, the 95% confidence limits of the probabilistic mean sensitivity are then 0.384 and 0.639.

Turning to the autocorrelation, time series’ variance, and process standard deviation, the resulting numerical values for our case study are *r*_*ac*1_ = −0.494 (without any unit), $s_{ts}^{2}=0.00989\;{\mathrm{cm}}^{2},$ and *s*_*proc*_ = 0.114 cm. The probabilistic parameters, adjusted for the uncertainty of ring boundaries, are ${\tilde{r}}_{ac1}=-0.490,$ ${\tilde{s}}_{ts}^{2}=0.00823\;{\mathrm{cm}}^{2},$ and ${\tilde{s}}_{proc}=0.104.$ The probabilistic parameters are always smaller, because at least some *P*_*i*_ and *P*_*i*+1_ are smaller than 1 in (6), (10), and (11). While the autocorrelations are between minus and plus one, the variances and standard deviations are always positive, but unbounded.

## Sensitivity analysis

How much does uncertainty about the exact input probabilities and locations of false and missing boundaries in a time series affect statistical age determination? In the above section on estimating the probabilities, we mentioned possible sources of uncertainty about them. There is also methodological uncertainty. For example, the result of classifying the recognizability of boundaries and crossdating with difficult-to-recognize boundaries can depend to a relatively large degree on the analyst. In that regard, dendrochronological analysis can be as much an art as a science. Some type of sensitivity analysis is highly recommendable, to evaluate how much the uncertainty and possible ranges of the probabilities of different ring boundary types affect estimated ages and growth curves.

For the expected age, Theorem 1 provides an easy way to judge the sensitivity of the expected age to changes in the input probabilities *P*_*i*_. In our case study, with 48 boundaries, each representing one year’s growth with input probability 0.83, the mean age at the 48th boundary is 0.83⋅48 = 39.84 years. If the 48 input probabilities increased, for example, from 0.83 to 0.95 (or 14.5%), the mean age would increase by 14.5% to 45.6 years. Thus, the mean age changes by the same proportion as that of the input probabilities, i.e. the probabilities that the boundaries are annual. As a consequence, at given radiuses for the analyzed boundaries the growth decelerates with increasing input probabilities (but the confidence curves get narrower, as explained below).

**Fig 6:**
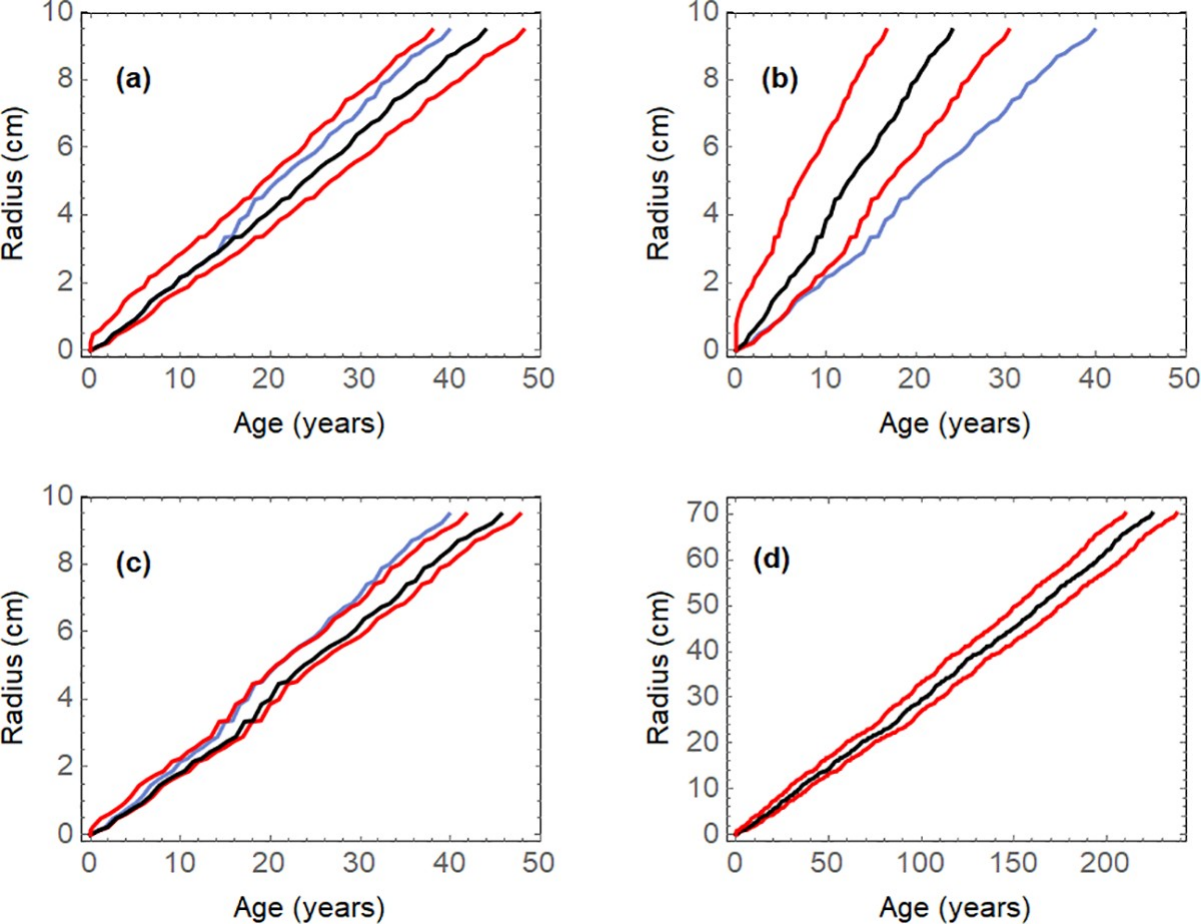
Simulations to analyze the sensitivity of the curves of radius as a function of expected age. (a) Four missing rings are assumed in the data of Fig 2; (b) The input probabilities are 0.5 instead of 0.83; (d) The input probabilities are 0.95 instead of 0.83; (d) Pseudorandom input probabilities between 0.5 and 1, and pseudorandom widths between 0 and 0.5 cm for 300 boundaries.

Fig 6 illustrates the sensitivity of the results to the input parameters in four simulations:

- **Four missing rings in the data of Fig 2:** In Fig 2 one notes some ring widths that seem unusually wide, and missing boundaries could be the reason. If one standardizes (“studentizes”) the 48 ring widths, by subtracting from each value the mean width and dividing by the standard deviation, the values can be compared against a *t*-distribution with 47 degrees of freedom. Four widths (numbers 17, 19, 21, 38) result as statistically significant outliers, whose probability is smaller than 5% to belong to the *t*-distribution. The missing boundaries are assumed to divide these rings of widest width in two equal halves, each with an input probability of 1, i.e., we assume to be certain about them. The curve of expected age decelerates, and becomes a little bit smoother. The maximum radius is reached 4 years later.
- **Input probabilities lower:** To consider a wide possible range of uncertainty about the true input probabilities, in this simulation *P*_*i*_ = 0.5 for *i* = 1 to 48, instead of 0.83 (39.8% lower). The curve of expected age accelerates, and the confidence interval becomes wider, because there is less certainty about a boundary to be annual. Technically, the variance decreases with increasing *P*_*i*+1_. This can be seen by applying (3) for the situation used in the above proof:
  $$\begin{array}{l} s_{{\bar{A}}_{i+1}}^{2}=t^{2}\cdot L_{i,t}\cdot \left(1-P_{i+1}\right)+{\left(t+1\right)}^{2}\cdot \left(L_{i,t+1}\cdot \left(1-P_{i+1}\right)+L_{i,t}\cdot P_{i+1}\right)+\\ \;{\left(t+2\right)}^{2}\cdot L_{i,t+1}\cdot P_{i+1}-{\left(t+L_{i,t+1}+P_{i+1}\right)}^{2}. \end{array}$$
  Taking the derivative with respect to *P*_*i*+1_, and simplifying results in
  $$ds_{{\bar{A}}_{i+1}}^{2}/dP_{i+1}=L_{i,t}+L_{i,t+1}+2\cdot t\cdot L_{i,t}+2\cdot t\cdot L_{i,t+1}-2\cdot \left(P_{i+1}+t\right).$$
  All variables are positive, so that an increase in *P*_*i*+1_ causes a decrease in the change of the variance $s_{{\bar{A}}_{i+1}}^{2},$ because of the term −2⋅(*P*_*i*+1_+*t*).
- **Input probabilities higher:** Now *P*_*i*_ = 0.95 for *i* = 1 to 48, instead of 0.83. The curve of expected age decelerates, and the confidence interval becomes narrower.
- **Random input probabilities and random widths over 300 boundaries:** The last simulation shows a longer time series, where the input probabilities and ring widths were chosen (pseudo)randomly, with input probabilities between 0.5 and 1, and widths between 0 and 0.5 cm. One sees that the confidence interval widens slightly with expected age, without any unreasonable behavior.

## Concluding remarks

For over a century, dendrochronology has operated with the goal of assigning an exact calendar year to each tree ring detected in the trunk wood. Once years have been assigned, statistical methods are generally used in dendrochronology to analyze the time-series of ring widths, for example to extract climate signals with as little noise as possible, noise that can be caused by different tree ages, tree-trunk geometry, and site differences [5]. While there is great value in meeting such a level of certainty [7], it is not always possible to know the exact calendar year when each growth ring was formed. In this context, it is valuable to develop a formal approach to statistical (i.e., probabilistic) age determination, which has never been introduced into the field. Here we offer such a statistical approach, assigning an input probability that each boundary is indeed an annual growth ring, and thus provide a formal way to treat each boundary with skepticism. A computer algorithm for facilitating all calculations in *Mathematica* is provided as supporting information.

Age and growth estimation of trees is basic to many fields, such as forest management and ecology [31], carbon stock analysis [32], forest restoration and conservation [33], and forest economics [34]. The uncertainty-intolerant approach would force one to discard many samples in which some ring boundaries are well marked, while others are doubtful. In tropical lowland forests with less pronounced annual climate seasonality, there is strong variation in recognizability, as well as variability in the timing of formation of boundaries [9], and crossdating may become impossible. Even in temperate zones, sampling focused on ecological questions in closed-canopy forests will lead to many increment cores that must be discarded under traditional standards for crossdating. In a study of *Acer saccharum* from Wisconsin (USA), crossdating results were inconclusive in 32% of the overtopped trees, because of low ring-width variability, multiple missing rings, and short duration of trouble-free segments [13]. Ecological sampling, particularly in tropical forests, will be important to quantify the role of forests in the global carbon cycle, and project future carbon storage in forests. These applications make the need for uncertainty quantification more pressing.

Finally, growth analysis based on annual marks is not only possible for trees. The methods of probabilistic age determination presented here could well be extended to study time series of growth marks in roots of perennial herbs [35], fish scales and otoliths, bivalve shells, corals, turtle scutes [36], bones of cervids and primates [37], and dental development data [38].

## Supporting information

S1 File The S1 File Statistical Age Determination.nb provides the computer code in a *Mathematica* notebook to generate the output, explained in the article, also for other data. The file cannot be run without the *Mathematica* software (www.wolfram.com/mathematica/), but we provide the PDF *S1* File *Statistical Age Determination*.*pdf* to be able to see the information.
(ZIP)

S2 File The sample input file with the data from Fig 2 is *S2 Alchornea Input*.*csv*. The files *S2a Alchornea Input with 4 missing boundaries*.*csv*, *S2b Alchornea Input with lower probability*.*csv*, *S2c Alchornea Input with higher probability*.*csv*, and *S2d 300 boundaries random*.*csv* contain the input for the sensitivity analyses, shown in Fig 6. (ZIP)
