# Supplementary material for: Statistical age determination of tree rings
Source: PLoS One. 2020 Sep 22;15(9):e0239052. doi: 10.1371/journal.pone.0239052 (PMC7508393; doi:10.1371/journal.pone.0239052)
Supplement: S1 File — The file cannot be run without the Mathematica software (www.wolfram.com/mathematica/), but we provide the PDF S1 File Statistical Age Determination.pdf to be able to see the information. (ZIP) [file pone.0239052.s001.zip › S1 Statistical age determination of tree rings.pdf]

```
Style[DateString[], 15, ColorData[59, 1], Bold, FontFamily → "Helvetica"]
```

Sun 9 Aug 2020 19:24:46

Computer program

Statistical age determination of tree rings

Mathematica notebook

Dr. Martin Ricker

mricker@ib.unam.mx, martin_tuxtlas@yahoo.com.mx

Input:

The notebook imports an *Excel* file of type “csv” (“comma delimited”); this type is automatically a single-sheet file, and the *Mathematica* import works better for large files than the xlsx type. The name has to be indicated in the following line:

```
inputFile = "S2 Alchornea Input.csv";
```

If the file is not in the current directory or cannot be found, then one has to provide the complete path of location (as given in the file properties).

The file consists of four columns and any number of rows (see sample file). The first line contains the column titles; the exact column titles don’t matter, because they are only for orientation, and are not used further. Of the four columns, only the second (radius increments) and fourth (probabilities) are used by the algorithm, while the other two columns contain auxiliary information for labeling the output. The four columns contain the following data:

1) The *boundary number* is typically an integer, though for missing boundaries, we use for example 10, 10.5, 11, where 10.5 refers to a missing boundary after boundary 10.

2) The *radius increment* in centimeters (cm), though the unit cm is only used for labeling. The increments should positive real number.

3) The *boundary type* indicates annual (probability = 1), recognizable (probability < 1) or missing boundaries. The column is only for orientation, and is not used further.

4) The *probabilities* should be positive real numbers larger than 0 and not larger than 1. For a recognizable boundary, the number represents the probability that a recognizable boundary is an annual, regular (and not a false) boundary. For a missing boundary, the number represents the probability that a missing (annual) boundary is expected at a specific location in a core sample.

The first row is the base boundary, numbered as boundary 0 and with a probability of 1 (recognizable and regular). It contains the increment of the first tree ring. The probabilities of the subsequent boundaries may either vary or all be the same. The last boundary of the sample is distinct in that it has no increment (0 cm).

To run the algorithm, one chooses with the mouse “Evaluation” and subsequently “Evaluate Notebook”. The following code generates the calculation and subsequently the output. The output is explained at the end of the notebook.

Use the data from an input file to calculate expected ages with confidence intervals, and related output:

```
(* The input file is imported from the directory of the Mathematica notebook *)
SetDirectory[NotebookDirectory[]];
originalData = Import[inputFile]
```

```
{{Boundary number, Increment (cm), BoundaRecognizabley type, Probability},
 {0, 0.124, Annual, 1}, {1, 0.099, Recognizable, 0.83}, {2, 0.256, Recognizable, 0.83},
 {3, 0.129, Recognizable, 0.83}, {4, 0.167, Recognizable, 0.83},
 {5, 0.148, Recognizable, 0.83}, {6, 0.22, Recognizable, 0.83},
 {7, 0.3, Recognizable, 0.83}, {8, 0.172, Recognizable, 0.83},
 {9, 0.143, Recognizable, 0.83}, {10, 0.119, Recognizable, 0.83},
 {11, 0.282, Recognizable, 0.83}, {12, 0.092, Recognizable, 0.83},
 {13, 0.196, Recognizable, 0.83}, {14, 0.131, Recognizable, 0.83},
 {15, 0.179, Recognizable, 0.83}, {16, 0.136, Recognizable, 0.83},
 {17, 0.444, Recognizable, 0.83}, {18, 0.034, Recognizable, 0.83},
 {19, 0.482, Recognizable, 0.83}, {20, 0.146, Recognizable, 0.83},
 {21, 0.458, Recognizable, 0.83}, {22, 0.067, Recognizable, 0.83},
 {23, 0.271, Recognizable, 0.83}, {24, 0.217, Recognizable, 0.83},
 {25, 0.16, Recognizable, 0.83}, {26, 0.219, Recognizable, 0.83},
 {27, 0.173, Recognizable, 0.83}, {28, 0.133, Recognizable, 0.83},
 {29, 0.149, Recognizable, 0.83}, {30, 0.213, Recognizable, 0.83},
 {31, 0.318, Recognizable, 0.83}, {32, 0.154, Recognizable, 0.83},
 {33, 0.194, Recognizable, 0.83}, {34, 0.103, Recognizable, 0.83},
 {35, 0.241, Recognizable, 0.83}, {36, 0.33, Recognizable, 0.83},
 {37, 0.094, Recognizable, 0.83}, {38, 0.384, Recognizable, 0.83},
 {39, 0.111, Recognizable, 0.83}, {40, 0.239, Recognizable, 0.83},
 {41, 0.196, Recognizable, 0.83}, {42, 0.267, Recognizable, 0.83},
 {43, 0.092, Recognizable, 0.83}, {44, 0.181, Recognizable, 0.83},
 {45, 0.115, Recognizable, 0.83}, {46, 0.136, Recognizable, 0.83},
 {47, 0.256, Recognizable, 0.83}, {48, 0, Recognizable, 0.83}}
```

```
data = SortBy[Drop[originalData, 1], First];
probabilities = Transpose[data][[4]];


(* Following, equation 1 is implemented in a shorter
 way for sequential calculation for all tree boundaries *)
calculatedProbsPerAge0 = {{-1.}, {1}}; (* Age, probability *)
calculatedProbsPerAge = calculatedProbsPerAge0;

ageResults =
  Table[
   If[probabilities[[i]] ≠ 1,
    newAges = Append[calculatedProbsPerAge[[1]], calculatedProbsPerAge[[1, -1]] + 1];
    newcalculatedProbs = Append[calculatedProbsPerAge[[2]] * (1 - probabilities[[i]]),
       0] + Prepend[calculatedProbsPerAge[[2]] * probabilities[[i]], 0],
    newAges = calculatedProbsPerAge[[1]] + 1;
    newcalculatedProbs = calculatedProbsPerAge[[2]]];

   calculatedProbsPerAge = {newAges, newcalculatedProbs},
   {i, 1, Length[data]}];


ageRanges =
  Table[
   MinMax[ageResults[[i, 1]]],
   {i, 1, Length[ageResults]}];


expectedAges =
  Table[
   Total[ageResults[[i, 1]] * ageResults[[i, 2]]],
   {i, 1, Length[ageResults]}];


variancesN =
  Table[
   {Total[ageResults[[i, 1]]^2 * ageResults[[i, 2]]] - expectedAges[[i]]^2,
    Length[ageResults[[i, 1]]]},
   {i, 1, Length[ageResults]}];

standardDeviations = Round[Sqrt[Transpose[variancesN][[1]]], 0.001];
```

```
confIntervals =
  Prepend[
   Table[
    input = ageResults[[i]];

    inversePLCDF = Interpolation[
      Transpose[{Accumulate[Rationalize[input[[2]], 10^-500]], input[[1]]}],
      InterpolationOrder → 1];

    {If[inversePLCDF[[1, 1, 1]] < 0.025, inversePLCDF[0.025], input[[1, 1]]],
     inversePLCDF[0.975]},
    {i, 2, Length[data]}], {0, 0}];



boundaryNumber = Transpose[data][[1]];
radiuses = Accumulate[Prepend[Drop[Transpose[data][[2]], -1], 0]];




(* Calculate the characteristics of confidence intervals for ages *)
meanConfIntervalBelow =
  Mean[Drop[expectedAges, 1] - Transpose[Drop[confIntervals, 1]][[1]]];

meanConfIntervalAbove =
  Mean[Transpose[Drop[confIntervals, 1]][[2]] - Drop[expectedAges, 1]];

meanConfIntervalWidth = meanConfIntervalBelow + meanConfIntervalAbove;

ratioBelowAbove = meanConfIntervalBelow / meanConfIntervalAbove;

meanConfIntervalIncrementPerBoundary =
  (confIntervals[[-1]][[2]] - confIntervals[[-1]][[1]]) / (Length[data] - 1);


(* Calculate mean sensitivity *)
widths = Drop[Transpose[data][[2]], -1];

ms = 1 / (Length[widths] - 1) * Total[
    Table[
     Abs[2 * (widths[[i + 1]] - widths[[i]]) / (widths[[i + 1]] + widths[[i]])],
     {i, 1, Length[widths] - 1}]];
```

```
(* Calculate probabilistic mean sensitivity *)
msP = 1 / (Length[widths] - 1) * Total[
    Table[
     probabilities[[i + 1]] *
      Abs[2 * (widths[[i + 1]] - widths[[i]]) / (widths[[i + 1]] + widths[[i]])],
     {i, 1, Length[widths] - 1}]];

varianceMsP = 1 / (Length[widths] - 1) * Total[
     Table[
      probabilities[[i + 1]] *
       (2 * (widths[[i + 1]] - widths[[i]]) / (widths[[i + 1]] + widths[[i]]))^2,
      {i, 1, Length[widths] - 1}]] - msP^2;

SEmsS = Sqrt[varianceMsP / Length[widths]];

lowerCL = msP - 1.96 * SEmsS;
upperCL = msP + 1.96 * SEmsS;


(* Calculate alternative indicators for mean sensitivity *)

(* first-order autorcorrelation coefficient *)
rac1 = Total[(Drop[widths, -1] - Mean[widths]) * (Drop[widths, 1] - Mean[widths])] /
   Total[(widths - Mean[widths])^2];

(* first-order autorcorrelation coefficient,
taking into account probabilities of boundary existence *)
rac1New = Total[Drop[probabilities, -2] *
     (Drop[widths, -1] - Mean[widths]) * (Drop[widths, 1] - Mean[widths])] /
   Total[Drop[probabilities, -1] * (widths - Mean[widths])^2];

(* time-series variance *)
s2 = Total[(widths - Mean[widths])^2] / Length[widths];

(* time-series variance, taking into account probabilities of boundary existence *)
s2New = Total[Drop[probabilities, -1] * (widths - Mean[widths])^2] / Length[widths];

(* process standard deviation of time-series *)
sProc = Sqrt[s2 / (1 - rac1^2)];

(* process standard deviation of time-series,
taking into account probabilities of boundary existence *)
sProcNew = Sqrt[s2New / (1 - rac1New^2)];
```

```
graph1 = Show[
   ListPlot[Transpose[ageResults[[-1]]], Filling -> Axis,
    PlotMarkers → {"●", 8}, PlotStyle → {Red}, PlotRange → Full],

   Frame → {{True, True}, {True, True}},
   FrameStyle → 12,
   FrameLabel → {{Style["Probability", Black, FontSize → 12], None},
     {Style["Age (years)", Black, FontSize → 12],
      Style["Probability density function of last boundary (" <>
        ToString[Length[probabilities] - 1] <> ")", ColorData[59, 1], FontSize → 16]}},
   ImageSize → 400, AspectRatio → 0.75, ImageMargins → 8];

graph2 = Show[
   ListLinePlot[Transpose[{Accumulate[Transpose[Transpose[ageResults[[-1]]]][[2]]],
      Transpose[Transpose[ageResults[[-1]]]][[1]]}], PlotStyle →
     {Red, AbsoluteThickness[1.3]}, PlotMarkers → {"●", 7}, PlotRange → Full],

   Axes -> False,
   Frame → {{True, True}, {True, True}},
   FrameStyle → 12,
   FrameLabel → {{Style["Age (years)", Black, FontSize → 12], None},
     {Style["Cumulative probability", Black, FontSize → 12],
      Style["Cumulative distribution function\nof last boundary (" <>
        ToString[Length[probabilities] - 1] <> ")", ColorData[59, 1], FontSize → 16]}},
   ImageSize → 400, AspectRatio → 0.75, ImageMargins → 8];
```

```
maxAgesOverBoundaryNumber =
  Transpose[{Table[i, {i, 0, Length[data] - 1}], Transpose[ageRanges][[2]]}];

upperConfLimitsOverBoundaryNumber =
  Transpose[{Table[i, {i, 0, Length[data] - 1}], Transpose[confIntervals][[2]]}];

expectedAgesOverBoundaryNumber =
  Transpose[{Table[i, {i, 0, Length[data] - 1}], expectedAges}];

lowerConfLimitsOverBoundaryNumber =
  Transpose[{Table[i, {i, 0, Length[data] - 1}], Transpose[confIntervals][[1]]}];

minAgesOverBoundaryNumber =
  Transpose[{Table[i, {i, 0, Length[data] - 1}], Transpose[ageRanges][[1]]}];

graph3 = Show[
   ListLinePlot[maxAgesOverBoundaryNumber,
    PlotStyle → {Black, AbsoluteThickness[0.8]}],

   ListPlot[upperConfLimitsOverBoundaryNumber,
    PlotStyle → {Red}, PlotMarkers → {"●", 7}],

   ListPlot[expectedAgesOverBoundaryNumber,
    PlotStyle → {Black}, PlotMarkers → {"○", 11}, PlotRange → Full],

   ListPlot[lowerConfLimitsOverBoundaryNumber,
    PlotStyle → {Red}, PlotMarkers → {"●", 7}],

   ListLinePlot[minAgesOverBoundaryNumber,
    PlotStyle → {Black, AbsoluteThickness[0.8]}],

   Frame → {{True, True}, {True, True}},
   FrameStyle → 14,
   FrameLabel → {{Style["Age (years)", Black, FontSize → 14], None},
     {Style["Boundary number", Black, FontSize → 14],
      Style["Expected age, 95% confidence interval,\nand possible age range for
          each boundary number", ColorData[59, 1], FontSize → 16]}},
   ImageSize → 400, AspectRatio → 0.75, ImageMargins → 8];
```

```
radiusOverMaxAges = Transpose[{Transpose[ageRanges][[2]], radiuses}];
radiusOverUpperConfLimits = Transpose[{Transpose[confIntervals][[2]], radiuses}];
radiusOverExpectedAges = Transpose[{expectedAges, radiuses}];
radiusOverLowerConfLimits = Transpose[{Transpose[confIntervals][[1]], radiuses}];
radiusOverMinAges = Transpose[{Transpose[ageRanges][[1]], radiuses}];

graph4 = Show[
   ListLinePlot[radiusOverMaxAges, PlotStyle → {Black, AbsoluteThickness[1]}],

   ListLinePlot[radiusOverUpperConfLimits, PlotStyle → {Red, AbsoluteThickness[1.8]}],

   ListLinePlot[radiusOverExpectedAges, PlotStyle → {Black, AbsoluteThickness[1.5]},
    PlotMarkers → {"○", 11}, PlotRange → Full],

   ListLinePlot[radiusOverLowerConfLimits, PlotStyle → {Red, AbsoluteThickness[1.8]}],

   ListLinePlot[radiusOverMinAges, PlotStyle → {Black, AbsoluteThickness[1]}],

   Frame → {{True, True}, {True, True}},
   FrameStyle → 14,
   FrameLabel → {{Style["Radius (cm)", Black, FontSize → 14], None},
     {Style["Age (years)", Black, FontSize → 14],
      Style["Radius as a function of expected age, 95% confidence\ncurves,
          and possible age range (with linear interpolation\nbetween
          points)", ColorData[59, 1], FontSize → 16]}},
   ImageSize → 400, AspectRatio → 0.75, ImageMargins → 8];


graph1
graph2
graph3
graph4

Print[];
TableForm[Transpose[
  Join[{boundaryNumber}, {Transpose[data][[3]]}, {probabilities}, {radiuses}, {Table[
     ToString[Round[ageRanges[[i, 1]]]] <> "-" <> ToString[Round[ageRanges[[i, 2]]]],
     {i, 1, Length[ageRanges]}]}]],
 TableHeadings → {None, {"Boundary\nnumber", "Boundary\ntype",
    "Assigned\nprobability", "Radius", "Range of\npossible ages"}}]

Print[];
TableForm[Transpose[Join[{boundaryNumber}, {expectedAges},
   {standardDeviations}, {Transpose[variancesN][[2]]}, Transpose[confIntervals]]],
 TableHeadings → {None, {"Boundary\nnumber", "Expected\nage",
    "Standard\ndeviation", "\!\(\*StyleBox[\"n\",FontSlant->\"Italic\"]\)",
```

```
    "Lower\n95% CL", "Upper\n95% CL"}}]


(* Information about confidence interval for ages *)
Print[];
Print[];
TableForm[
 {{meanConfIntervalWidth, ratioBelowAbove, meanConfIntervalIncrementPerBoundary}},
 TableHeadings → {None, {"Mean width\nof confidence\ninterval (years)",
    "Ratio between mean\nhalf-intervals below\nand above expected ages",
    "Mean increment of\nconfidence interval\nper boundary (years)"}}]

(* Information about mean sensitivity and related parameters *)
Print[];
Print[];
TableForm[{{ms, Length[widths]}},
 TableHeadings → {None, {"Classical mean\nsensitivity", "Number of\nwidths"}}]

Print[];
TableForm[{{msP, SEmsS, lowerCL, upperCL}},
 TableHeadings → {None, {"Probabilistic\nmean\nsensitivity", "\nStandard\nerror",
    "Lower 95%\nconfidence\nlimit", "Upper 95%\nconfidence\nlimit"}}]

Print[];
TableForm[{{sProc, rac1, s2}, {sProcNew, rac1New, s2New}},
 TableHeadings → {{"Classical", "Probabilistic"},
   {"Process standard\ndeviation of\ntime-series (cm)",
    "\nFirst-order\nautorcorrelation", "Variance\nof time\nseries(cm^2)"}}]


initialString = "Input file: " <> ToString[inputFile] <> ", " <> ToString[DateString[]];

(* Export output data *)
outputData = Transpose[
   Join[{boundaryNumber}, {Transpose[data][[3]]}, {probabilities}, {radiuses},
    {Transpose[ageRanges][[1]]}, {Transpose[ageRanges][[2]]}, {expectedAges},
    {standardDeviations}, {Transpose[variancesN][[2]]}, Transpose[confIntervals]]];

Print[];
Export["Probabilistic ages output.xlsx",
 Prepend[Prepend[outputData, {"Boundary number", "Boundary type",
    "Assigned probability", "Radius", "Minimum possible age", "Maximum possible age",
    "Expected age", "Standard deviation", "Number of possible ages",
    "Lower 95% confidence limit", "Upper 95% confidence limit"}], {initialString}]]
```

```
(* Export additional parameters *)
Export["Additional parameters.xlsx", {{initialString}, {},
  {"Number of boundaries:", Length[probabilities]},
  {"Last expected age (years):", expectedAges[[-1]]},
  {"Mean width of confidence interval (years):", meanConfIntervalWidth},
  {"Ratio between mean half-intervals below and above expected ages:",
   ratioBelowAbove}, {"Mean increment of confidence interval per boundary (years):",
   meanConfIntervalIncrementPerBoundary}, {}, {"Classical mean sensitivity:", ms},
  {"Number of widths for sensitivities:", Length[widths]}, {},
  {"Statistical mean sensitivity:", msP}, {"Standard error:", SEmsS},
  {"Lower 95% confidence limit:", lowerCL}, {"Upper 95% confidence limit:", upperCL},
  {}, {"Classical process standard deviation (cm):", sProc},
  {"Classical first-order autorcorrelation:", rac1},
  {"Classical variance of time series (cm^2):", s2}, {},
  {"Probabilistic process standard deviation (cm):", sProcNew},
  {"Probabilistic first-order autorcorrelation:", rac1New},
  {"Probabilistic variance of time series (cm^2):", s2New}}]
```

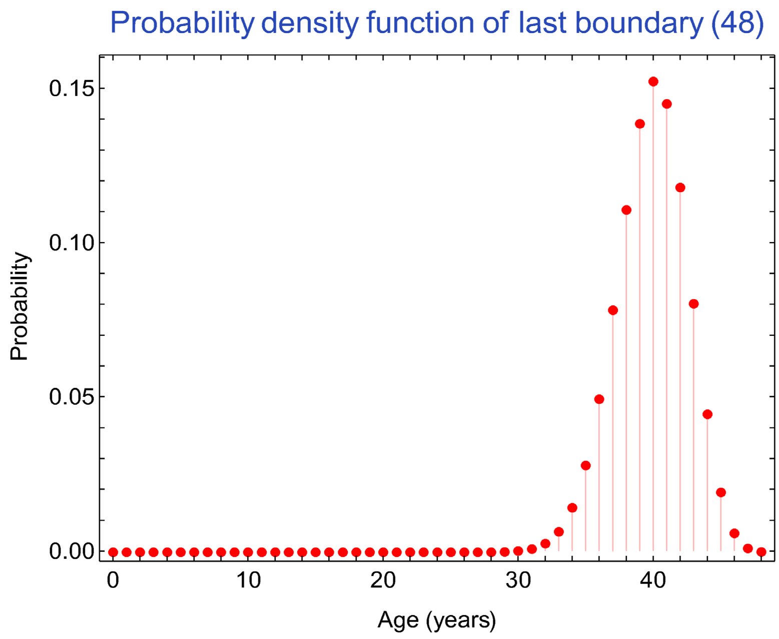

Cumulative distribution function
of last boundary (48)

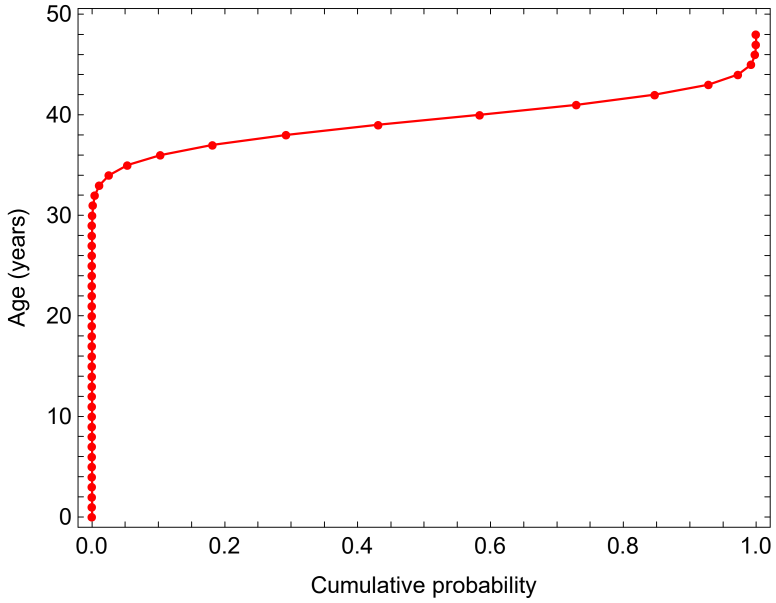

Expected age, 95% confidence interval,
and possible age range for each boundary number

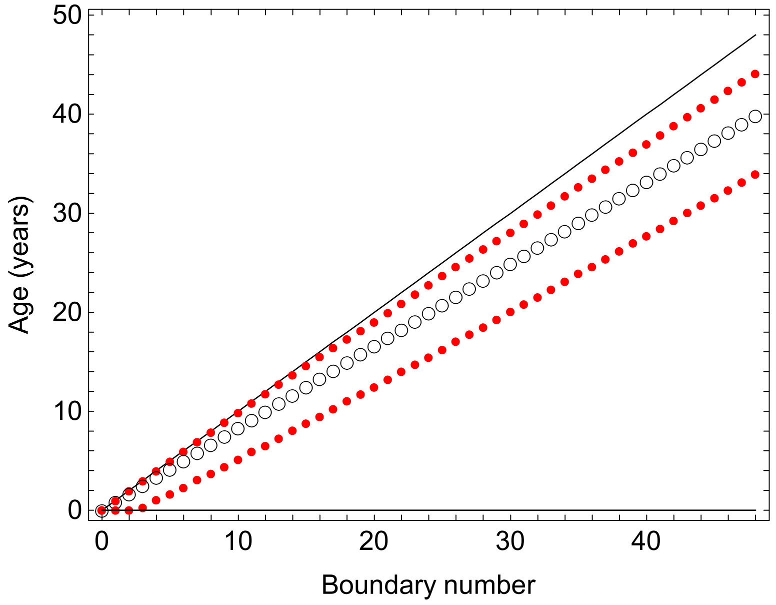

Radius as a function of expected age, 95% confidence curves, and possible age range (with linear interpolation between points)

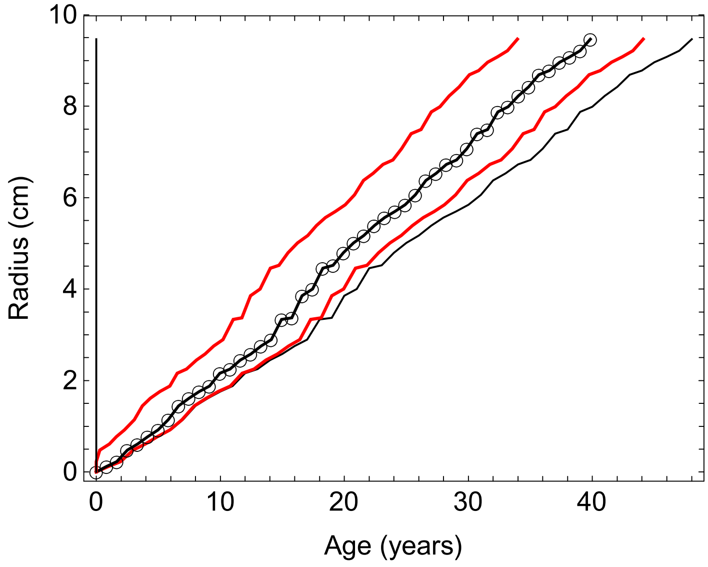

]//TableForm=

Boundary number	Boundary type	Assigned probability	Radius	Range of possible ages
0	Annual	1	0	0–0
1	Recognizable	0.83	0.124	0–1
2	Recognizable	0.83	0.223	0–2
3	Recognizable	0.83	0.479	0–3
4	Recognizable	0.83	0.608	0–4
5	Recognizable	0.83	0.775	0–5
6	Recognizable	0.83	0.923	0–6
7	Recognizable	0.83	1.143	0–7
8	Recognizable	0.83	1.443	0–8
9	Recognizable	0.83	1.615	0–9
10	Recognizable	0.83	1.758	0–10
11	Recognizable	0.83	1.877	0–11
12	Recognizable	0.83	2.159	0–12
13	Recognizable	0.83	2.251	0–13
14	Recognizable	0.83	2.447	0–14
15	Recognizable	0.83	2.578	0–15
16	Recognizable	0.83	2.757	0–16
17	Recognizable	0.83	2.893	0–17
18	Recognizable	0.83	3.337	0–18
19	Recognizable	0.83	3.371	0–19
20	Recognizable	0.83	3.853	0–20
21	Recognizable	0.83	3.999	0–21
22	Recognizable	0.83	4.457	0–22

23	Recognizable	0.83	4.524	0–23
24	Recognizable	0.83	4.795	0–24
25	Recognizable	0.83	5.012	0–25
26	Recognizable	0.83	5.172	0–26
27	Recognizable	0.83	5.391	0–27
28	Recognizable	0.83	5.564	0–28
29	Recognizable	0.83	5.697	0–29
30	Recognizable	0.83	5.846	0–30
31	Recognizable	0.83	6.059	0–31
32	Recognizable	0.83	6.377	0–32
33	Recognizable	0.83	6.531	0–33
34	Recognizable	0.83	6.725	0–34
35	Recognizable	0.83	6.828	0–35
36	Recognizable	0.83	7.069	0–36
37	Recognizable	0.83	7.399	0–37
38	Recognizable	0.83	7.493	0–38
39	Recognizable	0.83	7.877	0–39
40	Recognizable	0.83	7.988	0–40
41	Recognizable	0.83	8.227	0–41
42	Recognizable	0.83	8.423	0–42
43	Recognizable	0.83	8.69	0–43
44	Recognizable	0.83	8.782	0–44
45	Recognizable	0.83	8.963	0–45
46	Recognizable	0.83	9.078	0–46
47	Recognizable	0.83	9.214	0–47
48	Recognizable	0.83	9.47	0–48

]//TableForm=

Boundary number	Expected age	Standard deviation	*n*	Lower 95% CL	Upper 95% CL
0	0.	0.	1	0	0
1	0.83	0.376	2	0.	0.96988
2	1.66	0.531	3	0.	1.96371
3	2.49	0.651	4	0.279137	2.95628
4	3.32	0.751	5	1.06575	3.94732
5	4.15	0.84	6	1.63204	4.93653
6	4.98	0.92	7	2.27834	5.92353
7	5.81	0.994	8	3.07459	6.90787
8	6.64	1.062	9	3.71938	7.889
9	7.47	1.127	10	4.36772	8.86627
10	8.3	1.188	11	5.14293	9.83888
11	9.13	1.246	12	5.92762	10.8059
12	9.96	1.301	13	6.53247	11.7661
13	10.79	1.354	14	7.26874	12.7182
14	11.62	1.405	15	8.07648	13.6605
15	12.45	1.455	16	8.77995	14.591
16	13.28	1.503	17	9.4593	15.5072
17	14.11	1.549	18	10.2255	16.4062

18	14.94	1.594	19	11.0446	17.2846
19	15.77	1.637	20	11.7282	18.1381
20	16.6	1.68	21	12.4372	18.9906
21	17.43	1.721	22	13.2146	19.9416
22	18.26	1.762	23	14.0366	20.8874
23	19.09	1.801	24	14.7271	21.8268
24	19.92	1.84	25	15.4475	22.7583
25	20.75	1.878	26	16.2274	23.6804
26	21.58	1.915	27	17.0478	24.5913
27	22.41	1.952	28	17.7605	25.4888
28	23.24	1.988	29	18.4827	26.3704
29	24.07	2.023	30	19.2599	27.2332
30	24.9	2.057	31	20.0758	28.0736
31	25.73	2.091	32	20.8212	28.9634
32	26.56	2.125	33	21.5394	29.8962
33	27.39	2.158	34	22.3102	30.8219
34	28.22	2.19	35	23.1192	31.7392
35	29.05	2.222	36	23.9062	32.6466
36	29.88	2.254	37	24.6159	33.5423
37	30.71	2.285	38	25.3775	34.4243
38	31.54	2.316	39	26.1777	35.2902
39	32.37	2.346	40	27.0066	36.1374
40	33.2	2.376	41	27.7117	36.9856
41	34.03	2.405	42	28.4616	37.9108
42	34.86	2.434	43	29.251	38.8288
43	35.69	2.463	44	30.0703	39.7383
44	36.52	2.492	45	30.8268	40.638
45	37.35	2.52	46	31.5627	41.5263
46	38.18	2.548	47	32.3394	42.4013
47	39.01	2.575	48	33.1477	43.2609
48	39.84	2.602	49	33.9617	44.1027

]//TableForm=

Mean width of confidence interval (years)	Ratio between mean half−intervals below and above expected ages	Mean increment of confidence interval per boundary (years)
6.92181	1.60191	0.21127

]//TableForm=

Classical mean sensitivity	Number of widths
0.616237	48

]//TableForm=

Probabilistic mean sensitivity	Standard error	Lower 95% confidence limit	Upper 95% confidence limit
0.511476	0.0651653	0.383752	0.6392

]//TableForm=

	Process standard deviation of time-series (cm)	First-order autorcorrelation	Variance of time series(cm^2)
Classical	0.114436	-0.494396	0.00989462
Probabilistic	0.104089	-0.490154	0.00823156

Probabilistic ages output.xlsx

Additional parameters.xlsx

Output:

The output, presented above and in two *Excel* output files, starts with four graphs showing

1) the probability density function of the last boundary;

2) the cumulative distribution function of the last boundary;

3) the expected age (black circles), its 95% confidence intervals (red points), as well as the possible age range (as black lines), each as function of the boundary number (the count of the boundary number here is independent of the indicated boundary number in the first column of the input file); and

4) the estimated growth curve of radius as a function of expected age (black line with circles), with 95% confidence curves (red lines), and the possible age range (outer black lines), with linear interpolation between points.

Next, two tables are shows. The first table presents five columns: the boundary number, the boundary type, and the assigned probability (all from the input file), the radius calculated cumulatively from the input increments, and the resulting range of possible ages for each boundary number. The second table repeats the boundary number, followed by the expected age, its standard deviation, the corresponding number of ages from which the two previous parameters were calculated, and the lower and upper 95% confidence limits. If the lower 95% confidence limit does not exist at early ages, then the lower age limit is given. All parameters are given from the base boundary to the last boundary. All numbers are given with up to six significant digits.

The tables are followed by three parameters that characterize the 95% confidence intervals:

1) The *mean width of the confidence intervals* is calculated by taking the mean of the confidence

intervals for all boundaries except the base boundary (which has a confidence interval of 0 years).

2) The *ratio between the mean half-intervals below and above expected ages* is the mean of all half-interval widths below the expected age, divided by the mean of all half-interval widths above the expected age.

3) The *mean increment of the confidence interval per boundary* is the width of the last confidence interval (i.e., of the last boundary), which is the widest, divided by the number of increments (i.e., the number of boundaries minus 1).

Finally, the classical mean sensitivity is shown (with the number of widths for its calculation), followed by the probabilistic mean sensitivity (with its standard error, and confidence intervals), and the process standard deviation in classical and probabilistic form (with the corresponding first-order autocorrelations and time series' variance).

In addition, all numerical values of the output (used also for drawing the graphs) are stored in two *Excel* files in the current directory. The two files are called "Probabilistic ages output.xlsx" (with the data from the two tables) and "Additional parameters.xlsx" (with all additional data). Note that files with these same name are overwritten without any warning (unless they are open). Also note that more decimals of the provided numbers are given in these output files than in the above presentation.
